# Vulnerability of small-scale fishers to benzene exposure and the current knowledge gap on benzene-exposure in Brazilian fishers

**DOI:** 10.7717/peerj.7483

**Published:** 2019-08-13

**Authors:** Rachel Ann Hauser-Davis

**Affiliations:** Laboratório de Avaliação e Promoção da Saúde Ambiental, IOC, Oswaldo Cruz Foundation, Rio de Janeiro, Brazil

**Keywords:** Fishers, Vulnerability, Small-scale fisheries, Brazil, Benzene

## Abstract

Fishers are a particularly vulnerable population, chronically exposed to many stresses, injuries and health conditions directly linked to their fishing activities. This includes benzene exposure through gasoline and diesel exhaust fumes. Benzene is a known carcinogen, and has been assessed in many worker groups, but reports on fisher benzene exposure are extremely scarce in the literature. This paper discusses benzene exposure in small-scale fishers and reflects on the current knowledge gap on benzene-exposure in Brazilian fishers.

## Introduction

Fishers are a particularly vulnerable population, as they have, increasingly, suffered from a broad array of complex environmental, social, economic, and political pressures and changes worldwide (Chen & Lopez-Carr, 2015). These include, but are not limited to, climate change and environmental degradation. These factors are extremely significant, as they directly affect fish populations, which are the usually the only source of income for this group (Chen & Lopez-Carr, 2015).

In addition, this group is also exposed to many stresses, injuries and health conditions directly linked to their fishing activities, such as musculoskeletal stress, acute injuries and chemical exposure (Kirrane et al., 2007; Marshall et al., 2004). These are considered chronic conditions as many small-scale commercial fishers worldwide begin fishing as teenagers and continue well into their sixties or seventies (IBGE, 2017; Thummachinda et al., 2002). Thus, fishery activities are regarded as one of the most dangerous occupations worldwide (Jensen, 1997; Matheson et al., 2001; Oldenburg, Harth & Manuwald, 2015).

Fishers are also exposed to a wide range of chemicals involved in boat and gear maintenance and use (Kirrane et al., 2007; Marshall et al., 2004). These include several carcinogenic chemicals present in gasoline and diesel exhaust fumes, such as polycyclic aromatic hydrocarbons (PAHs) and other carbons, like benzene, toluene and xylene (Oldenburg, Harth & Manuwald, 2015). Among these, benzene has been reported as of significant concern. In this context, this paper discusses benzene exposure in small-scale fishers and reflects on the current knowledge gap on benzene-exposure in Brazilian fishers.

## Survey Methodology

The survey methodology applied herein comprised searches using the Web of Science, Pubmed, Google Scholar, Scielo and Scopus (Elsevier) scientific databases, from January to February 2019. The terms “Benzen* AND Fisher* OR Fishermen OR Fisherwomen” were used for the search, and only results in the “article” category, in English, were considered. All publishing years were taken into account. The conducted search resulted in very few hits. Because of this, references contained within the returned results were assessed concerning inclusion in this study, and further searches were carried out at the Google search engine using more general search terms, to broaden the search, as follows: words “Benzene”, “Fisher”, “Fishermen”, “Fisherwomen”, “S-phenylmercapturic acid”, “*trans, trans*-muconic acid” and “leukemia”

## Results

The scienciometric search resulted in very few hits, corroborating that assessments on benzene contamination in fisher populations are extremely scarce. After the scientific database and Google search engine assessments, the following papers of interest specifically on the subject of benzene exposure in fishers were obtained (*n* = 7), displayed in Table 1.

## Discussion

### Benzene exposure effects and biomarkers

Benzene is present in petroleum and petroleum-derived products and is generated by the incomplete combustion of organic matter (Fernandes et al., 2002). It is highly volatile and exposure occurs mostly though inhalation, with about 50 to over 90% of inhaled benzene being absorbed (Ong & Lee, 1994). As it is a carcinogenic substance, no safe level of exposure is recommended (WHO, 2010).

**Table 1:** Results of the search for articles on benzene exposure on fishers (scientific database and Google search engine assessments).

**Year**	**Authors**	**Title**	**Journal**
2018	Fiebelkorn, S., Meredith, C	Estimation of the leukemia risk in human populations exposed to benzene from tobacco smoke using epidemiological data	Risk Analysis
2017	Moraes, E. S., et al.,	Analysis of individuals with leukemia: Cancer surveillance system limitations.	Ciência & Saúde Coletiva
2015	Oldenburg, M., et al.	Comparison of hospitalization among German coastal and deep sea fishermen.	International Archives of Occupational and Environmental Health
2014	Tsai, R. J., et al.	Acute myeloid leukemia risk by industry and occupation	Leukemia & Lymphoma
2013	Arnold, S.M., et al.	The use of biomonitoring data in exposure and human health risk assessment: benzene case study	Critical Reviews in Toxicology
2007	Kirrane, E., et al.,	Personal exposure to benzene from fuel emissions among commercial fishers: Comparison of two-stroke, four-stroke and diesel engines.	Journal of Exposure Science and Environmental Epidemiology
2002	Thummachinda, S., et al.,	High urine ttMA levels among fishermen from a Thai rural villa	The Southeast Asian Journal of Tropical Medicine and Public Health

Acute benzene effects include narcosis: headache, dizziness, drowsiness, confusion, tremors and loss of consciousness, while its chronic effects comprise acute myeloid leukemia (acute non-lymphocytic leukemia) and aplastic anemia and evidence is available that is may also cause acute and chronic lymphocytic leukemia, non-Hodgkin’s lymphoma (NHL) and multiple myeloma (IARC, 1987). In addition, decreased host resistance to infection and chromosomal aberrations in both humans and animal studies have also been reported (WHO, 2010).

Several hematological parameters are altered in benzene-exposure scenarios, such as hematocrit, platelets, mean corpuscular volume, white and red blood cell counts, as well as absolute lymphocyte counts (Fan, 2014). However, these are all unspecific markers. Currently, only two specific validated benzene metabolite biomarkers are applied, urinary, trans, trans-muconic acid (t,t-MA) and S-phenylmercapturic acid (SPMA). Both are considered reliable biomarkers concerning occupational benzene exposure (ACGIH, 2017), as they have been shown to correlate well with external exposure to benzene in several studies carried out on occupationally-exposed subjects. However, some studies indicate that t,t-MA displays lower specificity than SPMA, due to relatively high background values of creatinine, which may be found in non-exposed individuals, and recommend its use only in higher benzene exposure scenarios (Boogaard & Van Sittert, 1996; Protano et al., 2012).

Some studies have also proposed associations between t,t-MA and hematological parameters, such as the study carried out by Wiwanitkit, Suwansaksri & Soogarun (2004), where correlations between t,t-MA and platelet count in subjects occupationally exposed to benzene were assessed. Although no significant correlations were observed, the authors indicated that platelet counts and plateletcrits (PCT) decreased while urinary t,t-MA increased, and that, when applying the upper normal limit t,t-MA level as the cutoff level, statistically significant lower platelet counts and PCT were observed in the subjects with urine t,t-MA higher than upper normal limit. This indicates that other benzene exposure biomarkers may arise in the future.

Benzene is also found in tobacco smoke, although the risk of leukemia from this type of lower environmental level exposure is still not fully understood (Snyder, 2014). However, while research results concerning this link are not always consistent, evidence of a positive association between these factors has been reported (Lichtman, 2007). In fact, recent studies estimating leukemia risks in human populations exposed to benzene from tobacco smoke using epidemiological data indicate that an increased risk of leukemia from low-level exposure to benzene seems to exist, and that benzene may contribute up to a third of smoking-induced leukemia (Fiebelkorn & Meredith, 2018).

Regarding tobacco smoking effects on benzene biomarkers, studies have indicated that direct effects on both SPMA and t,t-MA are observed in smokers compared to non-smokers. For example, one study involving 446 healthy volunteer residents from the general population since at least 10 years in an area of central Italy reported that SPMA concentrations in smokers (93) was about ten-fold higher than in non/ex-smokers (197/156), while smoker t,t-MA levels were about two-fold higher than in non/ex-smokers (Tranfo et al., 2017). Thus, tobacco smoking is a confounding variable regarding benzene exposure that should be taken into account in benzene assessments.

### Benzene exposure in fishers

Fishers are exposed to benzene by turning on their vessel engines and inhaling diesel exhaust fumes. Studies concerning benzene exposure in fishers are extremely scarce. In one report, high-performance liquid chromatography was applied to determine this biomarker in urine from 49 subjects (30 fishers and 19 control subjects), from a Thai rural community (Thummachinda et al., 2002). Mean urinary t,t-MA levels in fishers (0.180 ± 0.130 mg/g creatinine) were significantly higher than in the control group (0.015 ± 0.053 mg/g creatinine) (*p* < 0.05). The authors, thus, classified the study population at high risk due to chronic benzene exposure.

In another report, personal exposure to benzene in commercial fishers was compared among two-stroke, four-stroke and diesel engines (Kirrane et al., 2007). The authors employed passive monitors to determine benzene emissions in the three engine categories, and applied mixed-effect linear regression models to predict exposure levels. The predicted benzene exposure levels to fishers on boats equipped with two stroke, four-stroke and diesel engines were of 58.4, 38.9 and 15.7 mg/m3, respectively. The authors hypothesis, that fishers who use carbureted two-stroke engines were expected to present higher hydrocarbon exposure than those who use diesel engines or less polluting four-stroke engines, was, thus, confirmed, as two-stroke engines release high levels of hydrocarbons because oil is mixed with the fuel to lubricate the piston and the fuel intake and exhaust ports are open simultaneously during a portion of compression–combustion cycle (Kirrane et al., 2007).

Other studies carried out with this vulnerable group are not directly linked to benzene exposure, but to its main carcinogen effects (leukemia and NHL, among others) (Arnold, 2013). For example, one study carried out comparisons between hospitalization rates among German coastal and deep-sea fishers, using data from a health insurance company for seafarers, from 1997 to 2007 (Oldenburg, Harth & Manuwald, 2015). The results indicate that German fishers showed a considerably higher standardized hospitalization ratio (SHR) for malignant neoplasms, respiratory cancer, and for non-Hodgkin lymphoma (NHL). Comparing coastal and deep-sea fishers, the risk for respiratory cancer and NHL among coastal fishers exceeded that of deep-sea fishers, and differences between qualification levels among fishers were also observed, where less qualified deep sea fishers displayed considerably higher SHR for malignant neoplasms than more highly qualified ones.

In another study, significantly increased acute myeloid leukemia risks were noted for fishers and related fishing workers (Tsai et al., 2014), although no investigations on their causes were performed.

It is noteworthy that climate change effects, such as increasing sea temperature and ocean acidification, among others, has led to marked alterations in fish species distributions, both in latitude and depth, resulting in faster life cycles and smaller body sizes of many commercial species worldwide (Perry et al., 2005). This may, in turn, result in longer and higher amounts of fishing trips for fishers, due to higher search efforts for commercial species, which, in turn, may lead to increased benzene exposure. No studies correlating these factors, however, have been addressed in the literature.

### Benzene legislation in Brazil

In Brazil, benzene is considered a health risk factor due to occupational exposure since the 1930s. However, only from the 1980s did an intensification of trade union movements begin, claiming the social visibility of problems arising from the use of benzene (Brasil, 2018).

With the increase of the union pressure, public institutions began to carry out studies and epidemiological investigations in the work environments with benzene, culminating in Brazil’s Benzene Agreement, signed on September 28th, 1995 and the establishment of a Permanent National Commission on Benzene on March 26th, 1996, composed of a tripartite body formed to discuss, negotiate and monitor the agreement. The main responsibilities of this Commission are to complement the agreement concerning issues such as changes in worker health status and propose and monitor studies, research, scientific events and changes in legal instruments (De Freitas, Porto & Machado, 2006).

The Benzene Agreement includes the creation of certification for the controlled use of this substance, the establishment of penalties for non-adherents, decreased benzene content in gasoline to a maximum of 1% and an agreement to decrease benzene content in manufactured products to 0.1% at most by 2007. The agreement also establishes muconic acid as the biological benzene exposure indicator (De Freitas, Porto & Machado, 2006).

However, despite the existence of indicators of environmental benzene targets in the air, such as the Technological Reference Value - Time Weighted Average (VRT-MPT) as a mandatory element to guide the continuous control programs and better occupational conditions (where the benzene concentration limit for the environment is set at 2.5 ppm (8.1 mg/m3) for steel companies and 1.0 ppm (3.3 mg/m3) for other companies that produce, or manipulate benzene and its liquid mixtures with 1% or more of its volume) (Graciani & Ferreira, 2014), it is not possible to correlate their values with health risks. Therefore, the Brazilian Legislation on Benzene indicates that contact with benzene should be avoided and that there is no safe level of occupational exposure to this substance (Brasil, 2018). This is in agreement with WHO guidelines (WHO, 2010), which indicate that, as benzene is a carcinogenic substance, no safe level of exposure is recommended, so even low levels should be investigated.

### Studies associating benzene exposure and fishery activities in Brazil

Awareness of public health impacts resulting from benzene exposure to benzene is increasing in developing countries, although few have introduced policies and regulations in this regard (Thummachinda et al., 2002). Brazil is an exception, since benzene is considered a priority for the Brazilian National Health System (SUS) (Moura-Correa & Santana, 2014). A National Health Surveillance program (VISAT) has been implemented, aiming at the development of intervention policies regarding risk factors and determinants in worker health hazards, which includes benzene exposure (Moura-Correa et al., 2014). However, although benzene is targeted by health surveillance in Brazil, scarce data are available on both occupational or environmental benzene exposure (Moura-Correa & Santana, 2014).

In addition, few studies focus on understanding fisher organization and working conditions, and even less so concerning chemical exposure, are available for Brazil (Nogueira, De Souza & Brígida, 2017) and, to the best of our knowledge, no benzene studies in this regard are available for this country.

As in other countries, small-scale fishers in Brazil secure a large share of the domestic supply of fishery products, usually using low-impact technologies, with almost no public support (Nogueira, De Souza & Brígida, 2017). Over 60% of Brazil’s total fish landings originate from artisanal fisheries (FAO, 2010). In 2011, a total of 875 700 fishers (both men and women participate in fishery activities in Brazil, where one in three fishers is a woman) were directly engaged in full time fishing activities, and Brazil’s fishing fleet comprised over 60 000 vessels, with about one third being motorized (FAO, 2010).

Professional Brazilian fisher age distribution indicates that most range from 30 to 39 years old, totaling 242,683 workers, corresponding to 28.44% of the country’s total. The second age group with the highest number of fishers ranges from 40 to 49 years old, at 220,443 individuals, 25.84% of the national total. A significant amount of fishers comprises the age group between 50 and 59 years old, at 158,201 individuals, and between 20 and 29 years old, at 187,984, accounting for 18.54% and 22.03%, respectively, of country’s total fishers in the country (Nogueira, De Souza & Brígida, 2017).

Studies in Brazil indicate that fishers may work for over 40 years in fishery activities (Machado & Piccolo, 2018), and a recent census (IBGE, 2017; ND Mais, 2011) indicates that fishers from Santa Catarina, one of the Brazilian states with the most expressive fishing activities, represent 20% of people over 60 still in activity, alongside farmers, aquaculture and forest sector personnel, which, together, account for other 42 thousand people in this state only Thus, it seems likely that a significant amount of Brazilian fishers work up to or over 60 years old. Regarding working hours, informal assessments indicate from 12 to 14 h a day regularly, for both male and female fishers (CPP, 2017; ND Mais, 2011), and one report in the scientific literature indicates a means of 19.8 h a day in 2013 (Campos & Chaves, 2016). Thus many older fishers remain in activity, chronically exposed to exhaust fumes in their daily fishing activities for hours at a time.

Some studies have reported report leukemia incidences in this group cases in several Brazilian regions, although encompassing agricultural, hunting and fishing workers as a single group (Moraes et al., 2017). In addition, incomplete occupational information in Brazilian cancer hospital records is a reality, which directly reflects surveillance actions and associations between occupational, environmental and occupational exposure (Moraes et al., 2017), thus impeding further analyses of benzene-exposure scenarios concerning Brazilian fishers.

Furthermore, the impact of smoking habits in fishers, although of paramount importance, cannot be adequately evaluated, as no systematic review on the subject is available, and data is extremely discrepant throughout the different Brazilian regions. For example, previous studies in artisanal fisher communities in Brazil indicate that, from a sample universe of 18 individuals in the Copacabana artisanal fishing community in Southeastern Brazil, 33% continued to smoke or had been a smoker (ranging from 6 to 30 years of smoking), and that 67% broke the habit after 20–50 years of smoking, while from a total of 14 individuals, in the Paraty artisanal fishing community, also in Southeastern Brazil, 64% were or had been smokers (ranging from 13 to 65 years of smoking) (Cavichiolo, Begossi & Gurgel, 2013). On the other hand, in other fisher communities, the opposite is noted, i.e., in one artisanal fishing community in the Brazilian Amazon, in the Northwest region of the country, of a total of 517 participants, most (77.7%) reported not smoking at all, while 3.1% reported smoking one cigarette a day (Nuernberg, 2010). This further indicates that Brazilian fisher benzene-exposure scenarios are understudied with regard to several epidemiological factors, in addition to exposure during fisher activities.

The low number of papers found on the subject of fisher benzene exposure is expected, due to knowledge gap in this regard, reiterated by the present assessment. Other mini-reviews on other fisher related health assessments, such as fisher mortality, have demonstrated similarly low number of studies (nine) (Matheson et al., 2001).

## Conclusions

It is clear that fishers are a vulnerable group in many dimensions, i.e., social, health, environmental and economic. However, benzene exposure studies concerning this group are extremely scarce in the literature in general, and more so in Brazil, although the country has a vast fisher workforce. In addition, no systematic studies on the laboral characteristics of these workers (hours worked per day, maximum worked years) are available in the country, which makes it even more difficult to assess the incidence of benzene-related effects in this group. As these workers are in everyday contact with benzene, due to the fuel combustion used in their fishing activities, and, thus, chronically exposed to this carcinogenic pollutant, surveillance actions are paramount, regarding both benzene exposure and urinary biomarker monitoring, in order to implement decision-making and regulatory actions. It is important to note, however, that SPMA and t,t-MA analyses are considered costly in Brazil, due to expensive reagents such as clean-up cartridges and the use of high performance and expensive equipment, like LC-MS/MS and HPLC. Thus, in Brazil’s reality, few health surveillance laboratories are qualified to perform these analyses. In this context, estimating the costs of these technologies is paramount in order to aid in the development of better strategies to enable wider access to these analyses. These calculations should include standard operating procedures, and both direct (reagents, supplies, hired personnel, equipment depreciation, general expenses, among others) and indirect costs (such as cleaning, building maintenance, property security, and energy). In addition, as many products for these analyses must be imported, the value must be converted into Brazilian currency at the commercial exchange rate on the date of the proposal (which is, currently (2019) at a 4 to 1 ratio, in American dollars, which makes some items prohibitively expensive), and about 30% extra on the total value is applied to cover additional import costs. These actions can help to standardize these assessments in future studies concerning the inclusion of these tests as a routine for monitoring fisher health through biomarker analyses, with possible future implementation in the Brazilian Public Health System.

## Additional Information and Declarations

### Data Availability

The following information was supplied regarding data availability: This is a review article, no raw data was collected.
